# Characterization of chimeric antigen receptor modified T cells expressing scFv-IL-13Rα2 after radiolabeling with ^89^Zirconium oxine for PET imaging

**DOI:** 10.1186/s12967-023-04142-2

**Published:** 2023-06-07

**Authors:** Pamela Leland, Dhiraj Kumar, Sridhar Nimmagadda, Steven R. Bauer, Raj K. Puri, Bharat H. Joshi

**Affiliations:** 1 Tumor Vaccines and Biotechnology Branch, Division of Cellular and Gene Therapies, Office of Tissues and Advance Therapies, Center for Biologics Evaluation and Research, US Food and Drug Administration, 10903 New Hampshire Avenue, Silver Spring, MD 20993 USA; 2 Department of Radiology and Radiological Science, Johns Hopkins University School of Medicine, Baltimore, MD USA; 3 Present Address: Wake Forest Institute of Regenerative Medicine, Winston Salem, North Caroline USA; 4 Present Address: Iovance Biotherapeutics, San Carlos, CA USA

**Keywords:** CAR-T Cells, ^89^Zr, Radioactivity, PET

## Abstract

**Background:**

Chimeric antigen receptor (CAR) T cell therapy is an exciting cell-based cancer immunotherapy. Unfortunately, CAR-T cell therapy is associated with serious toxicities such as cytokine release syndrome (CRS) and neurotoxicity. The mechanism of these serious adverse events (SAEs) and how homing, distribution and retention of CAR-T cells contribute to toxicities is not fully understood. Enabling in vitro methods to allow meaningful, sensitive in vivo biodistribution studies is needed to better understand CAR-T cell disposition and its relationship to both effectiveness and safety of these products.

**Methods:**

To determine if radiolabelling of CAR-T cells could support positron emission tomography (PET)-based biodistribution studies, we labeled IL-13Rα2 targeting scFv-IL-13Rα2-CAR-T cells (CAR-T cells) with ^89^Zirconium-oxine (^89^Zr-oxine) and characterized and compared their product attributes with non-labeled CAR-T cells. The ^89^Zr-oxine labeling conditions were optimized for incubation time, temperature, and use of serum for labeling. In addition, T cell subtype characterization and product attributes of radiolabeled CAR-T cells were studied to assess their overall quality including cell viability, proliferation, phenotype markers of T-cell activation and exhaustion, cytolytic activity and release of interferon-γ upon co-culture with IL-13Rα2 expressing glioma cells.

**Results:**

We observed that radiolabeling of CAR-T cells with ^89^Zr-oxine is quick, efficient, and radioactivity is retained in the cells for at least 8 days with minimal loss. Also, viability of radiolabeled CAR-T cells and subtypes such as CD4 + , CD8 + and scFV-IL-13Rα2 transgene positive T cell population were characterized and found similar to that of unlabeled cells as determined by TUNEL assay, caspase 3/7 enzyme and granzyme B activity assay. Moreover, there were no significant changes in T cell activation (CD24, CD44, CD69 and IFN-γ) or T cell exhaustion (PD-1, LAG-3 and TIM3) markers expression between radiolabeled and unlabeled CAR-T cells. In chemotaxis assays, migratory capability of radiolabeled CAR-T cells to IL-13Rα2Fc was similar to that of non-labeled cells.

**Conclusions:**

Importantly, radiolabeling has minimal impact on biological product attributes including potency of CAR-T cells towards IL-13Rα2 positive tumor cells but not IL-13Rα2 negative cells as measured by cytolytic activity and release of IFN-γ. Thus, IL-13Rα2 targeting CAR-T cells radiolabeled with ^89^Zr-oxine retain critical product attributes and suggest ^89^Zr-oxine radiolabeling of CAR-T cells may facilitate biodistribution and tissue trafficking studies in vivo using PET.

**Supplementary Information:**

The online version contains supplementary material available at 10.1186/s12967-023-04142-2.

## Background

The approach of genetically engineering T cells to express chimeric antigen receptors (CARs) specifically targeting and killing cancer cells has become an important immunotherapeutic approach in the treatment of cancer [1–10]. CAR-T cells initiate T cell–mediated immune responses as they have target specific antibodies or single chain variable fragment (scFv) of the antibody genetically engineered with the intracellular signaling domains of the T cell receptor and costimulatory molecules [11, 12]. Recent advances in adoptive immunotherapy of cancer have led to the Food and Drug Administration (FDA) approval of CAR T-cells targeting CD19 and B-cell maturation antigen (BCMA) for advanced B-cell malignancies or relapsed/refractory B-cell malignancies. These CAR-T therapies have shown complete response in a large cohort of patients [7, 9, 13–15]. However, these therapies are also associated with serious adverse events (SAE) such as cytokine release syndrome (CRS) and neurotoxicity. CRS is a common complication observed in large percentage of subjects and can range from mild to severe form. CRS is often managed using tocilizumab (anti-IL-6R antibody), anakinra (anti-IL-1R antibody) or steroids but warrants a careful monitoring of the subjects under medical supervision and management. Neurotoxicity, which can be lethal, is another adverse event seen in a subset of patients [16–18]. Although it is believed that the cytokines and factors released by CAR-T cells and dying tumor cells may be contributing partly to these effects [19–24], the pathogenicity of these serious adverse events is not completely understood. Therefore, an understanding of the biodistribution and persistence of CAR-T cells in various tissues and vital organs, and their relationship to observed toxicities could provide insights into CAR T-cell related toxicities.

Molecular imaging can noninvasively monitor binding, trafficking, biodistribution and persistence of CAR-T cells which could improve our understanding of the dynamics of mechanism(s) underlying CRS, neurological and other toxicities. Radiolabeled cells can be visualized in vivo with very high signal-to-noise ratios by using single photon emission computed tomography (SPECT) and positron emission tomography (PET). Since it is desired to have lower radiation exposure while still obtaining high sensitivity, resolution, specificity, and sufficient duration to track the cells over multiple days, a long-lived positron-emitting radioisotope is needed. Zirconium-89 (^89^Zr) is a cyclotron-produced PET isotope with a half-life of 3.27 days and is routinely used to radiolabel large molecules including cells [25]. In the present study, we have used ^89^Zr-oxine to radiolabel the IL-13Rα2 specific CAR-T cells and tested their biological quality attributes in three naturally occurring IL-13Rα2 positive and one IL-13Rα2 negative glioma cell lines. We have optimized the labeling conditions by assessing key biological attributes of labeled CAR-T cells to determine the effects of ^89^Zr-radilabelling on the biological characteristics of these cells. The labeled CAR-T cells retain their critical biological functions and characteristics and thus would be suitable for future trafficking and biodistribution studies in vivo in animal studies.

## Materials and methods

### Synthesis of radiolabeled [^89^Zr]Zr-oxine

[^89^Zr]Zr-oxalate solution in 1.0 M oxalic acid was purchased from Washington University, St. Louis. [^89^Zr]Zr-oxalate was loaded onto an activated Waters Sep-pak QMA cartridge, washed with 10 ml water and eluted with 1.0 M HCl (aq). [^89^Zr]Zr-oxine complex was generated by conjugating oxine to [^89^Zr] ZrCl_4_ at room temperature. Oxine in chloroform (1 mg/mL, 500 µL) and ^89^ZrCl_4_ were mixed in the presence of 10 µL of Tween-80 and pH of the reaction was adjusted to 7.0 to 7.3 with 1 M Na_2_CO_3_. The reaction vial was vortexed to facilitate phase transfer and the two phases were allowed to separate (7–10 min). The chloroform phase was carefully extracted with a pipette and transferred to a separate vial. The pooled extract from multiple chloroform extractions was evaporated at 50 ºC under a stream of Argon. The residue containing ^89^Zr (oxinate)_4_ was re-constituted in 10% ethanol in PBS and sterile filtered. Purified and reconstituted ^89^Zr (oxinate)_4_ was analyzed on silica gel impregnated ITLC strips with ethyl acetate as mobile phase. The developed strips were cut and counted in an automated gamma counter. The radiochemical yields were 60.18 ± 19.20% (n = 8) with a radiochemical purity > 95%. The reference compound, Zr (oxinate)_4_ was synthesized according to reported procedure [26] and data (MS and ^1^H NMR) was consistent with the product formation.

### Cell cultures and generation of scFv-IL-13Rα2-CAR-T cells

Jurkat-T cells, A172, U87MG and T98G glioma cells lines were obtained from ATCC and grown as per the supplier’s instructions. U251 glioma cell line was obtained from NCI and maintained in RPMI complete medium with 10% FBS. T98G, A172 and U87MG glioma cell lines were maintained in RPMI or EMEM complete medium supplemented with 10% FBS. We have previously characterized these cell lines for IL-13Rα2 expression by RT-PCR for mRNA and ICC analyses for protein expression as described [27]. Human PBMCs were isolated from buffy coats of normal healthy blood donors who donated blood at the Division of Transfusion Medicine, NIH.

IL13Rα2-CAR T cells (termed CAR-T cell) were generated from CD4 and CD8 + ve T cells isolated from normal human blood donor buffy coat using Ficol-Paque plus density gradient technique (Cat# 17,144,002, Cytiva Life sciences, Marlborough, MA), activated with Dynabeads human T cell activator CD3/CD28 for T cell expansion and activation (Cat# 11131D, Thermo Fisher Scientific, Waltham, MA) and transduced with different multiplicity of infection (m.o.i) as described elsewhere (Joshi et al., unpublished observations). Briefly, a third-generation CAR construct consisting of single chain Fv (scFv) antibody sequence against IL-13Rα2 antibody (as ectodomain) and a CD28 transmembrane domain along with (CD3ζ) and (CD28 and or 41BB) endodomain sequences were placed into pCDH-MSCV-MCS-EF1-copGFP-T2A-Puro lentiviral vector (Cat# CD713B-1, System Biosciences, Palo Alto, CA) and packaged into 293 T cells by co-transfecting with three helper plasmids (pRRE, pRev and pCMV-VSV-G) to produce self-inactivating (SIN) lentiviral vector expressing CAR as generated IL13Rα2-CAR pseudo-lentivector. Jurkat-T cells were used as a control in parallel to assess the efficiency of transduction using similar m.o.i, and expansion and final manufacturing of CAR-T cells.

For CAR T cell expansion, the cells were activated with Dynabeads Human T-activator CD3/CD28 for T cell expansion and activation (Cat# 11131D, ThermoFisher Scientific, ThermoFisher Scientific, Waltham, MA) at a ratio of 3:1 (T cell to bead) and genetically modified via lentiviral transduction. T cells were maintained in culture at 0.6–1 × 10^6^ cells/mL in *T cell Transact* supplemented with 50 ng/ml IL-2 (cat# 130–111-160, Miltenyi Biotec, Waltham, MA).

### Radiolabeling of CAR-T cells with ^89^Zr-Oxine and retention of radioactivity

^89^Zr-oxine solution (1 μCi-10 μCi) and 10^6^ cells in phosphate-buffered saline were incubated at room temperature for 10–60 min at 1:20 or 1:40 volume ratios. We compared radiolabeling of CAR-T cells in serum free medium or in complete culture medium at 37 °C, room temperature or 4 °C. In an independent set of experiments, we incubated 1 μCi of ^89^Zr-oxine solution with varying number (1 -10 × 10^6^) of CAR-T cells to determine the number of CAR-T cells that can be radiolabeled in 1 μCi of ^89^Zr-oxine. The cells were washed with complete medium without fetal calf serum twice and with phosphate-buffered saline once.

For retention experiments, ^89^Zr-Oxine labeled CAR-T cells (radiolabeled CAR T cells) were cultured in T cell medium and sampled at the indicated time points. Radioactivity within the cell pellet and supernatant was assayed in a gamma-counter (WizzardD2 Automatic Gamma counter, Perkin-Elmer, Waltham, MA) to determine retention of radioactivity. Cell-associated radioactivity was determined as the amount of radioactivity in the final cell pellet at the specified time point.

### Efflux of ^89^Zr-oxine from radiolabeled CAR-T cells

In a parallel study to determine cellular efflux of radioactivity, we cultured 0.25 × 10^6 89^Zr-labeled CAR-T cells in each well of a six-well culture plate. The medium was replaced with fresh medium daily for 7 days, and radioactivity in the replaced medium was counted in a gamma counter. Three independent experiments were performed in quadruple runs and the results were expressed as mean ± SD.

### Cell viability and proliferation of Radiolabeled CAR-T cells

For all experiments, we examined the cell viability before labeling, after labeling, and at the indicated time points in culture by trypan blue exclusion technique. In an independent experiment, we incubated the cells with 1X PBS, which served as negative control. Each value is expressed as mean ± SD of three independent experiments performed in quadruplicate.

The effect of radiolabeling on CAR-T cellular proliferation was assessed by the CellTiter 96R AQuesous one solution (Cat# G3582, Promega, Madison, WI). Unlabeled and ^89^Zr-labeled CAR-T cells (2500 cells/well) were plated in quadruple wells of a 96-well culture plate and maintained at 37 °C in a CO_2_ incubator. Twenty microliters of MTS reagent were added in each well on day 3, 5 and 7 and number of proliferating cells in each well was quantified at 490 nm from absorbance of MTS formazon formed in presence of phenazine ethosulfate. Each value is expressed as mean ± SD of three independent experiments performed in quadruplicate.

### Caspase 3/7, Granzyme B activity and apoptosis in ^89^Zr-Oxine labeled CAR-T cells

We assessed the effect of radiolabeling on intracellular caspase 3/7 activity using earlytox Caspase-3/7 R110 assay kit following manufacturer’s recommendations (cat# R8346, Molecular Device, Sunnyvale, CA). Unlabeled cells served as control. A known number of unlabeled and ^89^Zr-oxine labeled CAR-T cells (200,000/well) were plated in a 6-well culture plate and maintained at 37 °C in a CO_2_ incubator. Intracellular caspase 3/7 activity from radiolabeled CAR-T cells was determined on day 3 and 7. The endpoint fluorescence was measured on a SpectraMax M5 plate reader (Molecular Device, San Jose, CA) at Ex/Em = 490/520 nm and each value are expressed as arbitrary fluorescence units.

In addition, we examined granzyme B activity from labeled and unlabeled CAR-T cell lysates on day 3 and 7 to assess any change introduced by radiolabeling of CAR-T cells using fluorometric assay kit following manufacturer’s instructions (cat# NBP2-54,853, Novus Biologicals, Bio-techne, R&D systems, Centennial, CO). The enzyme activity was determined using a non-fluorescent substrate, Ac-IEPD-AFC, which is hydrolyzed to produce AFC fluorescent product measured on a SpectraMax M5 plate reader (Molecular Device, San Jose, CA) (Ex/Em = 380 nm/500 nm) proportional to the granzyme B activity present in the CAR-T cell lysate. One unit of enzyme activity is defined as the unit that will cleave 1 pmol of the substrate per minute per mg protein at 37 °C. Both assays (Caspase 3/7 and Granzyme B) were performed in three independent experiments in quadruplicate and data were shown as mean ± SD.

The number of apoptotic nuclei were counted and quantified in quadruplicate on day 3 and 7. Briefly, 100,000 unlabeled and radiolabeled CAR-T cells were plated for 45 min in 4 well poly-L-lysine coated glass chambered slides and performed the assay using DeadEnd fluorometric TUNEL system as per manufacturer’s instructions (Cat# G3250, Promega Corporation, Madison, WI). After a brief wash with 1X PBS, 4 fields in each well were counted for 500 cells/field by three investigators in a blinded manner (a total of 2000 cells/well) for the TUNEL positive apoptotic cells. The assay was performed in three independent experiments in quadruplicate and data were shown as mean ± SD. [25].

### Assessment of T cell stimulation and T cell exhaustion biomarker expression in ^89^Zr-Oxine labeled CAR-T cells

Radiolabeled CAR-T cells were evaluated for their expression of CD44, CD25, CD69, and intracellular IFN-γ (Cat# 130–113-338, 130–115-535, 130–112-802 and 130–117-780; all antibodies from Miltenyi Biotec, Waltham, MA) as T cell activation markers after treating the cells with brefeldin A (Cat# 420,601, Bio Legend, San Diego, CA) by indirect immunofluorescence assay. Radiolabeled CAR-T cells were also examined for PD-1, LAG-3 and TIM3 expression by IFA on day 7 for T cell exhaustion biomarker expression (Cat# 130–116-683, 130–120-610, 130–106-432; Miltenyi Biotec, Waltham, MA).

Each value in both sets of T cell activation and exhaustion marker expression experiments is expressed as mean ± SD of four independent readings performed in quadruplicate for scoring in a blinded fashion for % positive cells expressing ≥ 2 + immunofluorescence intensity.

### Assessment of CAR-T cell subtype

Characterization of T cell subtype CD4, CD8, CD3 and scFV-IL-13Rα2 positive T cells in unlabeled and radiolabeled CAR-T cell product was examined by indirect immunofluorescence assay as described above (Cat# 130–114-531, 130–110-816, 130–113-138, Miltenyi Biotec, Waltham, MA). scFV-IL-13Rα2 expressing T cells were first incubated with IL-13R alpha 2Fc chimera (cat# 7147-IR-100, R&D Systems) after Biotinylating with Biotinylation kit (Cat# ab201796, abCam, Waltham, MA). Brilliant red fluorescence of Streptavidin–biotin conjugate was formed with Streptavidin alexa 546 (Cat# S11225, ThermoFisher Scientific, Waltham, MA). The slides were viewed under 200X magnification to count red fluorescent positive CAR-T cells. Each value in both sets of CAR-T cells is expressed as mean ± SD of four independent readings performed in quadruplicate for scoring in a blinded fashion for % positive cells expressing ≥ 2 + immunofluorescence intensity. Because of the technical challenges posed by radioactive samples, we performed a parallel experiment to compare the immunofluorescence assay with FACS assay using same antibodies and reagents, and assessed the concordance between these two assays, which was found to be > 94.0% (Additional file 2: Table S1).

### Analysis of migration potential of ^89^Zr-IL-13Rα2

The migration potential of radiolabeled IL13Rα2-CAR T cells was assessed in 24-well ChemoTx plates with a 5-μm pore diameter (Cat # ab235696, abCam, Cambridge, MA). In the lower chambers, 600 μL of unconditioned Dulbecco modified Eagle medium with 10, 50 and 1000 ng/ml hu-IL-13Rα2Fc (Cat# 7147-IR-100, R&D Systems, Minneapolis, MN) and 300 μL of either U251-or T98G tumor cell culture conditioned medium mixed with 300 μL of complete medium were added. The upper chambers were loaded with 500,000 CAR T cells/200 μl. After 6 and 20 h at 37 °C, residual cells were scraped off the polycarbonate filter, and the plate was centrifuged for 2 min at 400 × g. The filter was removed, and cells in the lower chamber were counted by trypan blue exclusion technique. Percentage migration was calculated as the number of cells in the lower chamber divided by the total number of cells plated per well. Each value is expressed as mean ± SD of three independent experiments performed in quadruplicate.

### Cytotoxic activity

We next performed an in vitro assay to determine the cell killing activity of non-labeled and radiolabeled CAR-T cells as effector cells by a robust homogeneous fluorescence-based non-isotopic cytotoxicity assay. The IL-13Rα2 positive (U251 and U87 MG) and IL-13Rα2 negative glioma tumor cells (T98G) as target cells are labeled by intracellular Calcein violet-acetoxymethyl ester (Cat# C34858, ThermoFisher Scientific, Waltham, MA) that has good retention in target cells [28, 29]. Release of Calcein Violet in the supernatants recovered at the end of 6 h of co-culture of target: effector cells in the ratio of 1:10, 1:20, 1:30, 1:40 and 1:50 is measured quantitatively on a fluorescent plate reader Spectramax M5 at Ex/Em = 400/452 nm). The data are shown as mean ± SD of three independent experiments performed in quadruplicate involving co-cultures of radiolabeled and unlabeled CAR-T cells.

### IFN-γ release

Non-labeled and radiolabeled IL13Rα2-CAR T (CAR-T) cells (100,000) were co-cultured for 20 h with equal number of IL-13Rα2 positive tumor cells in a well of 96 well round bottom plate. The cultures were centrifuged at 3500 × g for 10 min and supernatants were harvested for quantitative determination of IFN-γ secretion by ELISA assay (Cat# 430104, Bio legend, San Diego, CA). A plate-bound IL-13Rα2-Fc (R&D Systems, Minneapolis, MN; at 250, 500, 750 and 1,000 ng/well) was included as positive controls in the assay. Each value is expressed as mean ± SD of three independent experiments performed in quadruplicate.

### Statistical analysis

The data were compared using unpaired Student’s t-test analyses. *P* values less than 0.05, calculated by using GraphPad Prism software (Graph- Pad Software, La Jolla, Calif), were considered to indicate a significant difference. Two-way analysis of variance was used to compare labeling conditions (*n* = 4), the Wilcoxon test was used to obtain two-sided global *P *values for cell survival or proliferation (*n* = 4).

## Results

### Synthesis of ^89^Zr-oxine

We observed an average yield of 60.18 ± 19.20% (mean ± SD, n = 8) of ^89^Zr-oxine conjugate with radiochemical purity > 95% as investigated with chloroform extraction analyses. The ^89^Zr-oxinate_4_ was sterile filtered and used for subsequent experiments of cell labeling without further purification.

### Radiolabeling of CAR-T cells with ^89^Zr-oxine

We constructed a lentiviral vector encoding scFv derived from anti-IL-13Rα2 antibody, identified from a phage display library (Leland et al., unpublished data). Briefly, the construct contained a N-terminal leader sequence, a codon optimized transgene encoding anti-IL-13Rα2 scFv, a CD8α hinge region, a CD28 transmembrane domain, and signaling domains derived from CD28 cytoplasmic, 4-1BB co-stimulatory endodomains, and CD3ζ domain (Additional file 1: Figure S1). Using this construct, we manufactured CAR-T lentiviral vector after packaging into 293 T cells as described in Materials and Methods.

**Fig. 1:**
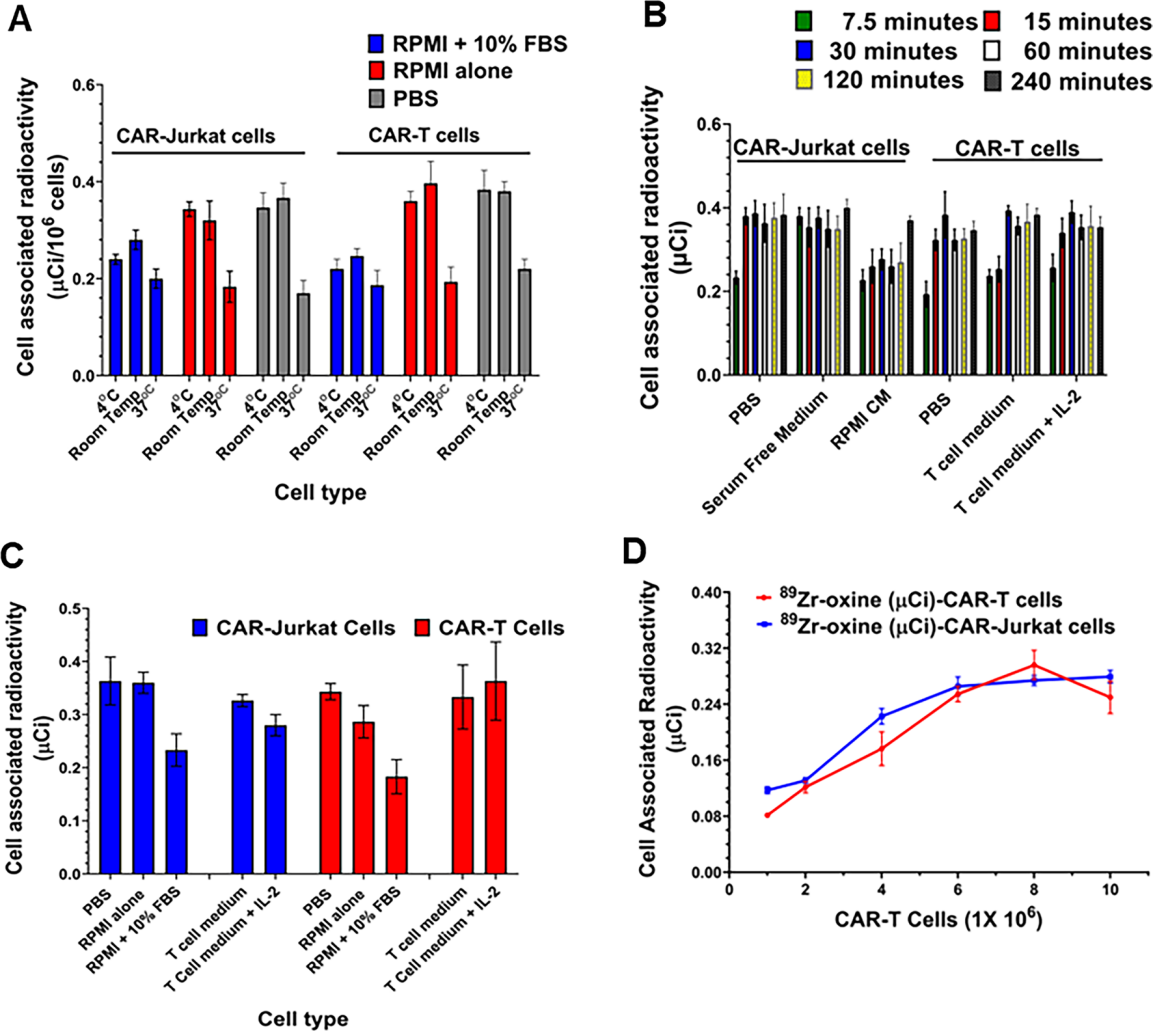
Radiolabeling of CAR-T cells with ^89^Zr-oxine **A** Effect of temperature on radiolabeling of CAR-Jurkat and CAR-T cells ranging from 4 °C to 37 °C. **B** Optimal time for radiolabeling of CAR-Jurkat and CAR-T cells performed at room temperature. **C** 1 × 10^6^ cells were radiolabeled with ^89^Zr-oxine in PBS, RPMI, RPMI with 10% FBS, T cell medium or T cell medium with IL-2 at room temperature. **D** ^89^Zr-oxine radiolabeled CAR-Jurkat and CAR-T cells retained cell associated radiolabel in a cell number dependent manner. Three independent experiments were performed in triplicate and results are expressed as mean ± SD

To determine the optimal labeling conditions, we compared cell labeling at 37 °C, room temperature, and 4 °C by using Jurkat and CAR-T cells in different medium. As shown in Fig. 1, the labeling at 37 °C was low under all buffer conditions (Fig. 1A). Maximum radiolabeling of Jurkat and CAR-T cells occurred in first 15 min at room temperature and then plateaued showing no significant change in values for next 6-h incubation (Fig. 1B). We observed that the highest radioactivity incorporation was achieved when the cells were labeled at room temperature or 4 °C in phosphate-buffered saline, serum free RPMI or T cell medium (≤ 0.35 μCi/10^6^ cells) compared to cells labeled at 37 °C (≤ [0.24 μCi/10^6^ cells] (Fig. 1A and C). Use of complete cell media at room temperature or 4 °C decreased the labeling efficiency to about 60%–76% of that in phosphate-buffered saline or serum free culture medium (*P* ≤ 0.05) (Fig. 1C). We also observed that 1.0 μCi of ^89^Zr-oxine could be used to efficiently radiolabel 5 × 10^6^ cells (Fig. 1D). Increasing the number cells beyond 5 × 10^6^ cells did not increase cell uptake of radioactivity in CAR-Jurkat or CAR-T cell under optimal radiolabeling conditions.

### Effect of radiolabeling with ^89^Zr-oxine on viability and proliferation of CAR-T cells

**Fig. 2:**
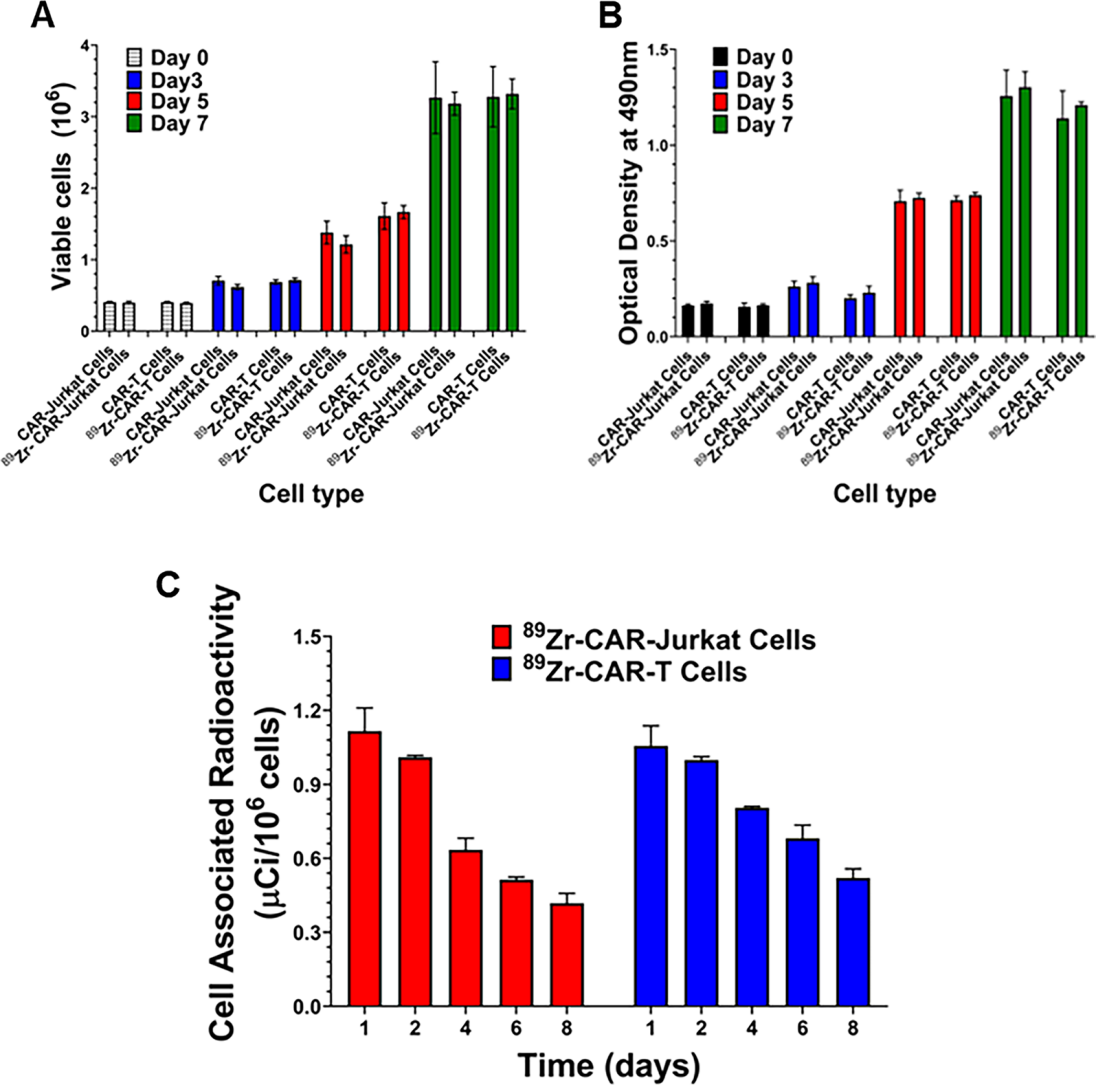
Effect of ^89^Zr-oxine on cell viability and proliferation of CAR-Jurkat and CAR-T cells. **A** Cell viability of radiolabeled and unlabeled CAR-Jurkat and CAR-T cells was determined at different time points by trypan blue exclusion technique. **B** Cell proliferation of radiolabeled and unlabeled CAR-Jurkat and CAR-T cells was determined at different time points by MTS cell proliferation assay. **C** Cell associated radioactivity was measured after spinning 1 × 10^6^ CAR Jurkat and CAR-T cells to determine retention of radioactivity at different time points. Three independent experiments were performed in triplicate and results were expressed as mean ± SD

Next, we determined if radiolabeling of CAR-Jurkat and CAR-T cells with ^89^Zr-oxine could exert any effect on cell viability and interfere with biological functions of radiolabeled CAR-T cells. As shown in Fig. 2A, we evaluated cell viability of CAR-Jurkat and CAR-T cells at different time points for 7 days after maintaining them in complete growth medium. No significant difference was observed in viable cell numbers between radiolabeled CAR-Jurkat or radiolabeled CAR-T cells and respective non-radiolabeled cells for 7 days post-labeling. We also examined for effects on metabolic and cell proliferation activity. A comparative study of cell proliferation between unlabeled and radiolabeled CAR-T cells showed that radiolabeled CAR-Jurkat or CAR-T cells showed no significant difference in proliferation rate from respective unlabeled cells when evaluated by the MTS cell proliferation assay (Fig. 2B). ^89^Zr labeled cells retained at least one third of radioactivity on day 8 (≥ 0.35 μCi/10^6^ cells) from initial value of on day 1 of culture (1.16 μCi/10^6^ cells) (Fig. 2C). The observed loss in cell associated activity on day 8 was found to be within predicted values after 2.5 half-life of isotope decay. This was further corroborated by our observations of radioactivity measured from cell free supernatants of radiolabeled CAR-T cells on a daily basis, which revealed no significant efflux of radioactivity for a period of 7 days. This confirmed that majority of the radioactivity was retained as cell associated radioactivity during the course of experiment. These data support the use of radiolabeled CAR-T cells for further in vivo evaluation.

### Characterization of T cell subtype of CAR-T cells after Radiolabeling with ^89^Zr-oxine

Next, we analyzed the subtypes of T cells for a comparative assessment and characterization of CD4 + , CD8 + , CD3 + and transgene expressing T cells in unlabeled and radiolabeled CAR-T cells as described in Materials and Methods. We observed that the radiolabeling of CAR-T cells with ^89^Zr-oxine had no significant effect on three major T cell subtypes of the cell product. Interestingly, the transgene expressing CAR-T cell population also showed no significant difference in % positive cells (number of cells expressing more than 2 + immunostaining intensity per 100 cells) before and after radiolabeling (Additional file 2: Table S2).

### ^89^Zr-oxine radiolabeling did not increase apoptosis in CAR-T cells

**Fig. 3:**
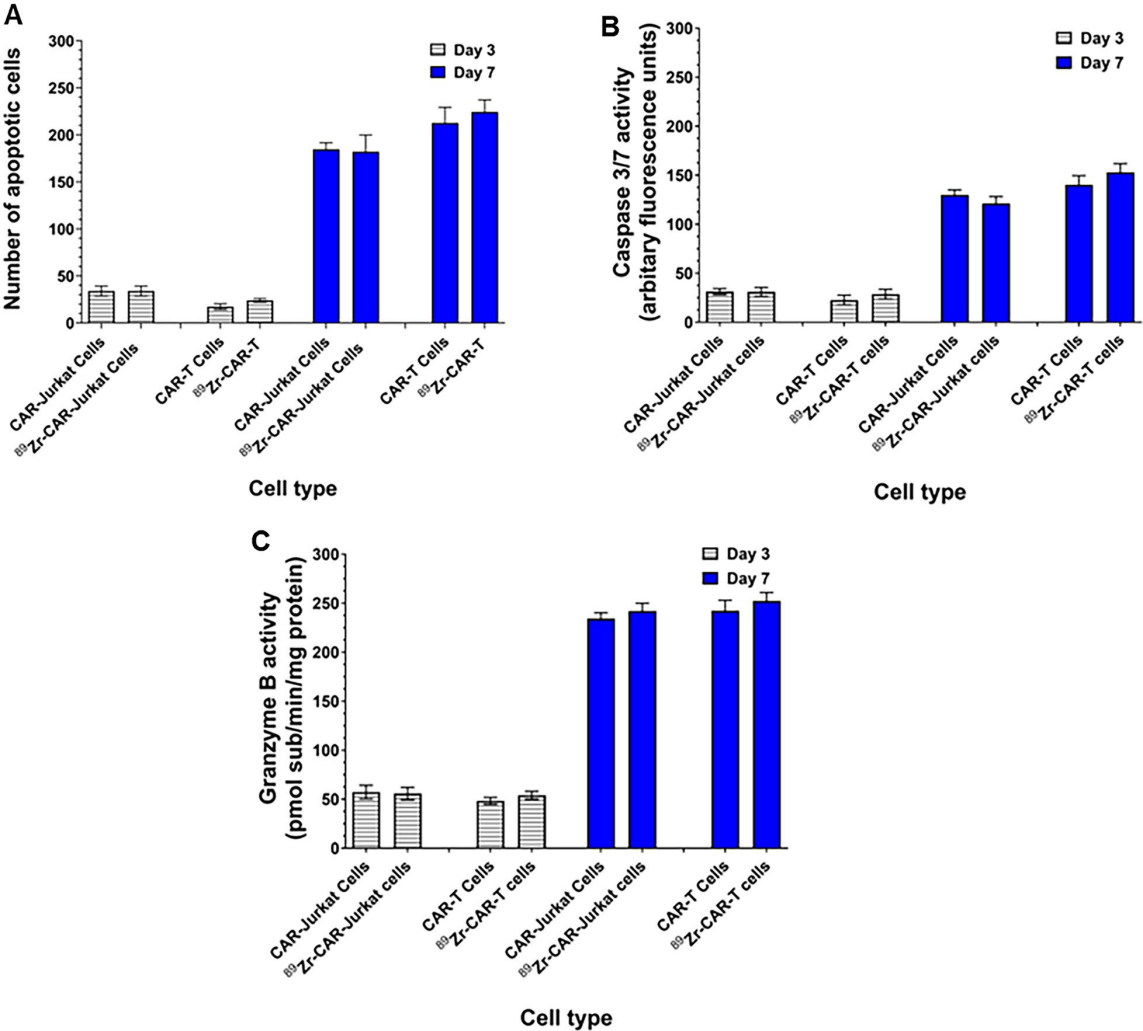
^89^Zr-oxine radiolabeling did not increase apoptosis in CAR-T Cells. **A** Number of apoptotic cells were counted in radiolabeled and unlabeled CAR-Jurkat and CAR-T cells on day 3 and day 7 as described in Materials and Methods. Each value represents a mean ± SD of three independent experiments performed in quadruplicate. **B** Caspase 3/7 activities from cell lysates obtained from unlabeled and radiolabeled CAR-Jurkat and CAR-T cells were measured by fluorometric assay on day 3 and day 7. Each value represents a mean ± SD of three independent experiments performed in quadruplicate. **C** Granzyme B activities from cell lysates obtained from unlabeled and radiolabeled CAR-Jurkat and CAR-T cells were measured by fluorometric assay on day 3 and day 7. Each value represents a mean ± SD of three independent experiments performed in quadruplicate

Unlabeled and ^89^Zr-oxine radiolabeled CAR-Jurkat and CAR-T cells were analyzed for apoptotic nuclei and Caspase-3/7 activities at day 3 and 7 of culture. As shown in Fig. 3A, no significant change in number of apoptotic nuclei was observed between labeled and non-labeled CAR-Jurkat or CAR-T cells on 3 and 7 time points. Also, ^89^Zr-oxine radiolabeling did not induce significant increase in Caspase-3/7 activity (Fig. 3B) or granzyme B activities in radiolabeled cells compared to unlabeled cells at day 3 or day 7 (Fig. 3C), which further corroborated the apoptosis assay results.

### ^89^Zr-oxine labeling did not alter CAR-T cell phenotype or function

**Fig. 4:**
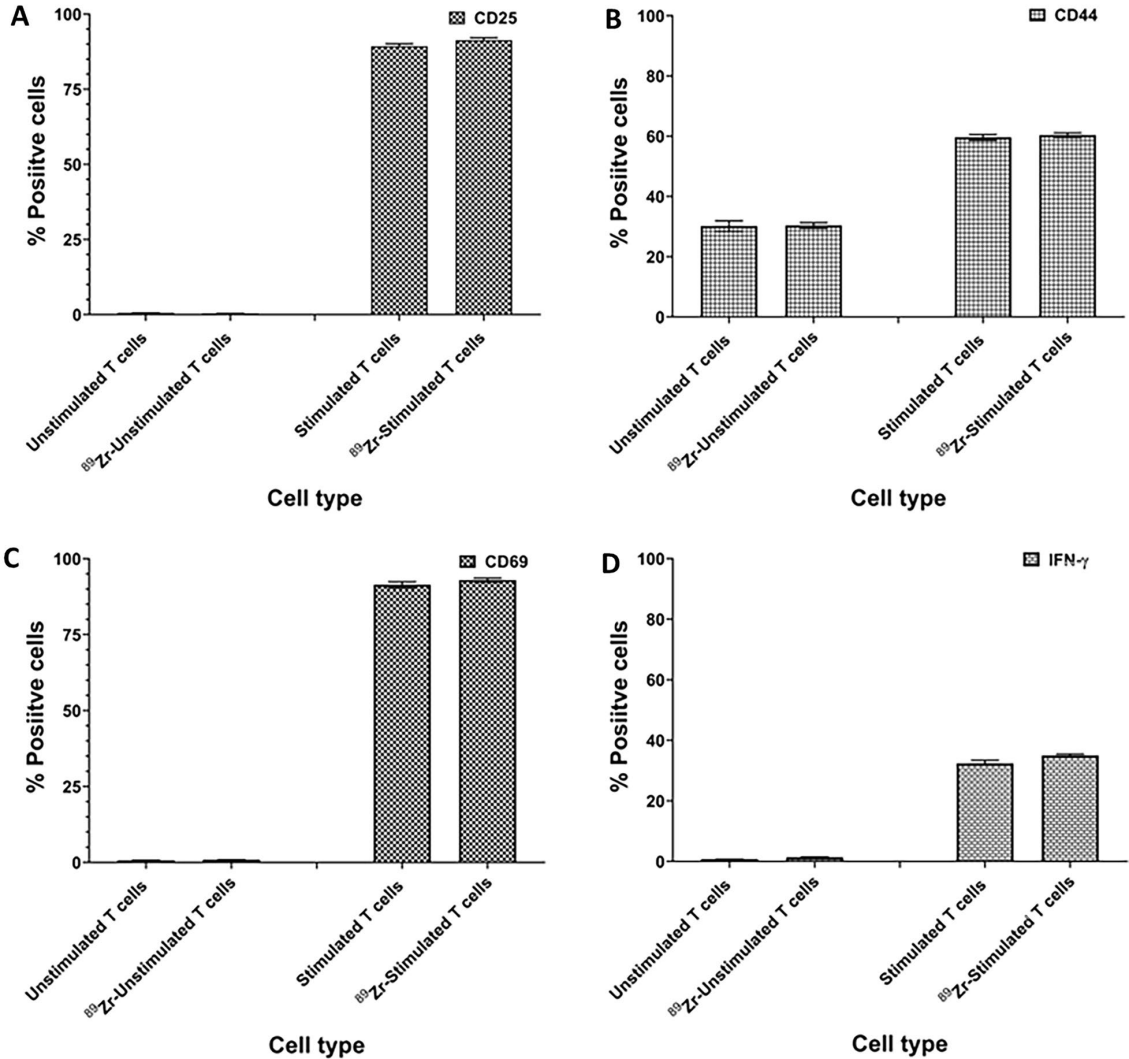
Radiolabeling with ^89^Zr-oxine did not alter phenotype and function of CAR-T cells. IFA data show ^89^Zr-oxine labeled and unlabeled CAR-T cells have no significant changes in **A** CD25, **B** CD44, **C** CD69 and **D** intracellular IFN-γ expression and the level of expression of all four markers was similar in ^89^ Zr-oxine labeled and unlabeled CAR-T cells. Representative data from one of three independent experiments is shown

As anticipated, we observed that stimulated T cells showed upregulated expression of CD25, CD44 and CD69 cell surface markers (Fig. 4A–C). As shown in Fig. 4D, stimulation of T cell with anti-CD3/CD28 antibody coated magnetic beads caused expression of intracellular IFN-γ. Radiolabeling of CAR-T cells with ^89^Zr-oxine did not change the expression of intracellular IFN-γ. These results suggest that the TCR stimulation of CAR-T cells was not affected by radiolabeling.

**Fig. 5:**
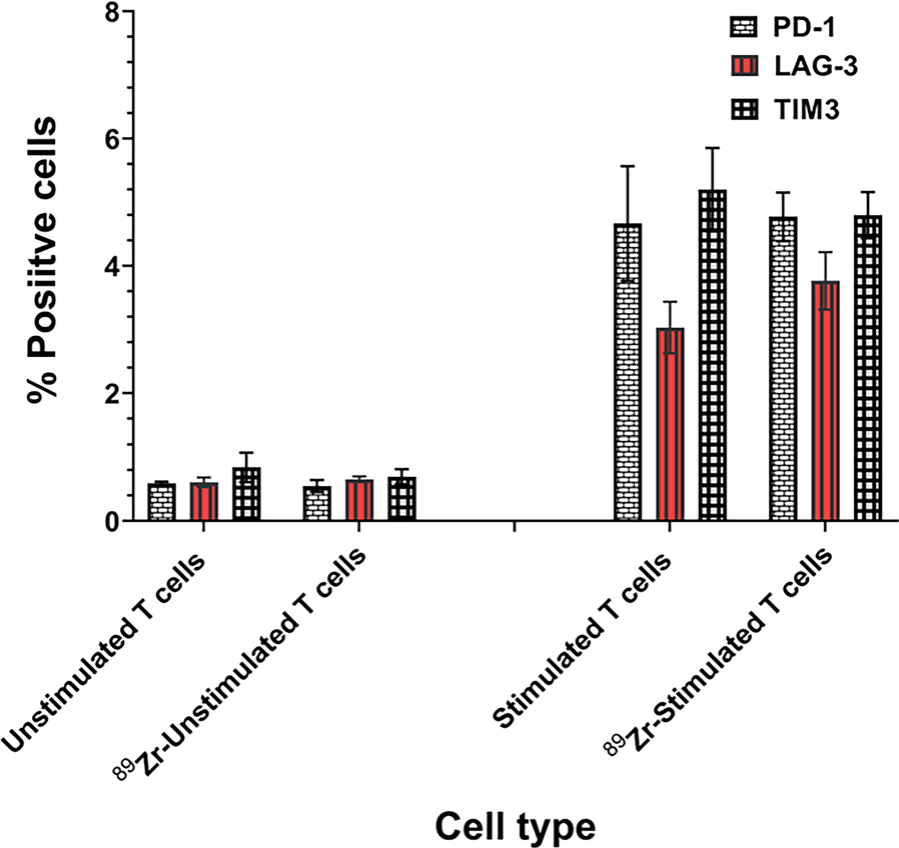
Radiolabeling with ^89^Zr-oxine did alter expression of T cell exhaustion markers in CAR-T cells. IFA analysis of ^89^Zr-oxine labeled and unlabeled CAR-T cells was performed for T cell exhaustion markers and no significant changes in PD-1, LAG-3 and TIM3 expression were noted. Representative data from three independent experiments is shown

To determine the impact of radiolabeling on T cell exhaustion, we examined the expression of three exhaustion markers on CAR-T cells. As shown in Fig. 5, ^89^Zr-oxine radiolabeling of CAR-T cells did not show any significant change in T cell exhaustion markers (PD-1, LAG-3 and TIM3) compared to unlabeled CAR-T cells, suggesting that the radiolabeled CAR-T cells maintained the phenotype.

### Effect of ^89^Zr-oxine labeling on migratory potential of CAR-T cells

**Fig. 6:**
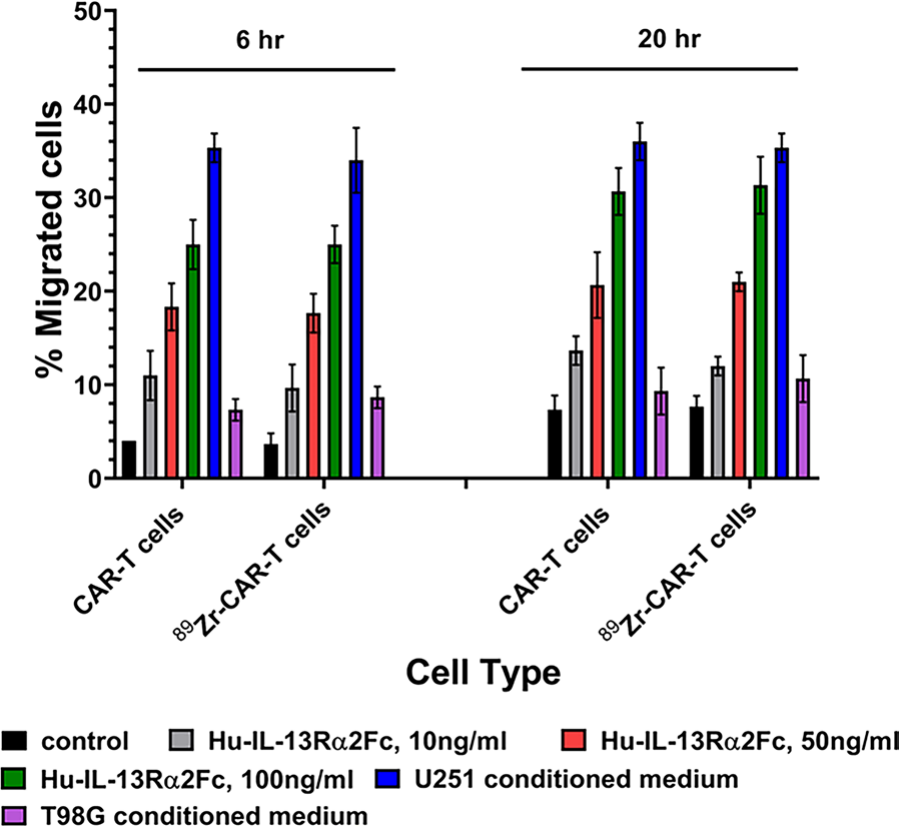
^89^Zr-oxine radiolabeling did not affect cell invasion ability of CAR-T cells. Cell invasion ability of radiolabeled and unlabeled CAR-T cells was unaltered at 6 h and 20 h time points in Boyden chamber cell migration assay in response to different concentrations of IL-13Rα2Fc chimeric protein. A representative data of three independent experiments performed in quadruplicate is shown

Next, we examined the effect of ^89^Zr-oxine radiolabeling on cell migration of CAR-T cells using a Boyden chamber assay to measure the response to IL-13Rα2Fc chimeric protein or conditioned medium obtained from IL-13Rα2 positive and IL-13Rα2 negative human glioma cell lines. As shown in Fig. 6, unlabeled and radiolabeled CAR-T cells migrated equally to human IL-13Rα2Fc in a concentration dependent manner at 6 and 20 h time points. Similarly, unlabeled and radiolabeled CAR-T cells migrated equally and migrated to conditioned medium from IL-13Rα2 positive glioma cells but not to condition medium from IL-13Rα2 negative glioma cells. Interestingly, there were a greater but non-significant number of CAR-T cells migrated to the lower chamber at 20-h time point. In contrast, no significant difference in cell migration was observed between unlabeled and radiolabeled CAR-T cells. A marginal increase in CAR-T cell migration to bottom chamber between 6 h and 20-h time points suggests that viable CAR-T cells continued growing during that time but non-significant differences in unlabeled and radiolabeled cell populations.

### Radiolabeled CAR-T cells-maintained potency

**Fig. 7.**
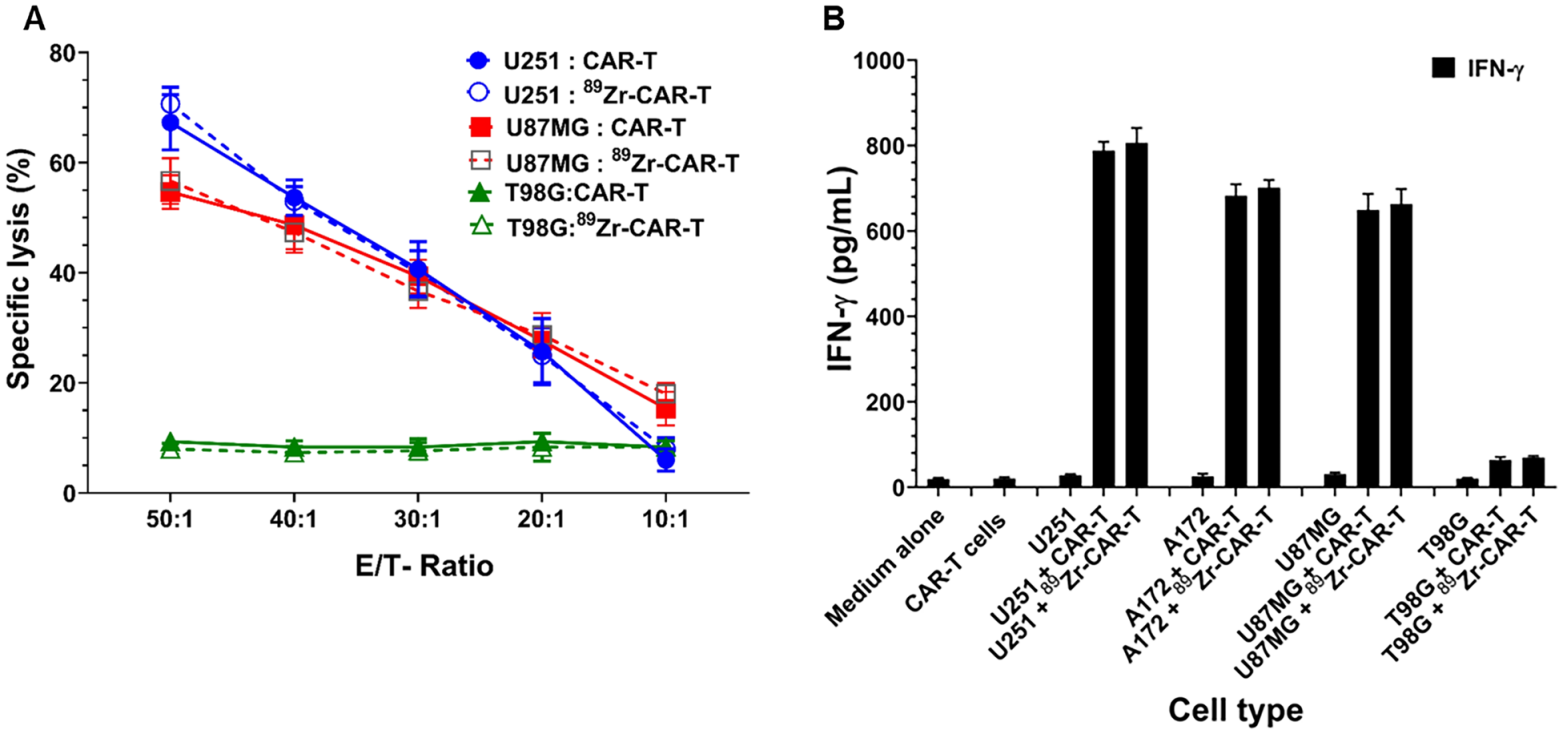
^89^Zr-oxine radiolabeling did not interfere with the biological functional potency of CAR-T cells. **A** In vitro potency assay of radiolabeled and unlabeled CAR-T cells showed no significant difference in their IL-13Rα2 positive target tumor cell killing abilities in a co-culture assay as described in Materials and Methods. **B** Co-culture assay of labeled and unlabeled CAR-T effector cells with IL-13Rα2 positive U251, A172 and U87MG target cells showed similar values of IFN-γ release in 20-h culture. IL-13Rα2 negative T98G glioma cells co-cultured with labeled and unlabeled CAR-T cells secreted basal amounts of IFN-γ. A representative data of three independent experiments performed in quadruplicate is shown

Next, we examined the potency of radiolabeled CAR-T cells against IL-13Rα2 positive U251 and U87 MG, and IL-13Rα2 negative T98G cells by a cell killing assay. Radiolabeled CAR-T cells mediated the cell killing of U251 and U87MG cells in effector cell number dependent manner similar to unlabeled CAR-T cells. In contrast, no cell killing was observed with IL-13Rα2 negative T98G glioma cells with non-labeled and radiolabeled CAR-T cells (Fig. 7A).

We also determined the potency of CAR-T cells by INF-γ release assay when CAR-T effector cells were co-cultured with IL-13Rα2 positive A172, U251 and U87MG cells and IL-13Rα2 negative T98G cells. CAR-T cells were co-cultured with equal numbers of target cells for 20 h. and IFN-γ measured in the supernatant by ELISA. As shown in Fig. 7B, the labeled and non-labeled CAR-T cells produced large and equal amount of IFN-γ in the supernatant when cultured with IL-13Rα2 positive glioma cell lines. In contrast, both labeled and non-labeled CAR-T cells secreted basal and minimal amounts of IFN-γ when cultured with IL-13Rα2 negative tumor cell line. Taken together, these data demonstrate that ^89^Zr-radiolabled IL-13Rα2 targeted CAR-T cells exhibit similar potency to that of non-labeled cells.

## Discussion

The primary objective of our present study is to provide proof-of-principle results by developing a highly sensitive, rapid and biologically functional cell population suitable for subsequent imaging studies to evaluate the biodistribution and trafficking of CAR-T cells in vivo in animal studies. Towards that goal, we have studied several crucial biological attributes of unlabeled and ^89^Zr radiolabeled CAR T cells in vitro to examine if radiolabeling process caused any significant interference in these biological quality attributes of CAR-T cells that are related to their functions in vivo. Maintaining these biological attributes after radiolabeling of CAR-T cells is of paramount importance to show that these cells may provide an integrated readout that is reflective of biodistribution and trafficking of functional and healthy CAR-T cells in vivo. The ability to trace radiolabeled CAR-T cells is of high importance for understanding the mechanism of action of efficacy and safety and may provide insight into particularly serious adverse events such as CRS and vital organ toxicities including neurotoxicity associated with this class of therapies.

The synthesis of ^89^Zr-oxine complex was accomplished with simple steps of mixing, which resulted in more than 60% of ^89^Zr being converted to the complex allowing us to add the resulting solution to the cell suspension for radiolabeling. Our optimization data for radiolabeling of CAR-T cells showed that the maximum radiolabeling occurred in 15 min of incubation at room temperature in serum free RPMI or T cell culture medium. A fixed amount of 1 μCi of ^89^Zr-oxine could optimally radiolabel ~ 4.5 × 10^6^ CAR-T cells demonstrating a linear cell associated retention of ^89^Zr-oxine with a labeling efficiency in the range of 30%. Since maximum labeling occurred at 4 °C and room temperature, we conclude that ^89^Zr-oxine complex does not require active cellular incorporation. Our data also confirm previously published results that ^89^Zr-oxine labeling does not depend on active cellular incorporation in vitro in the CAR-T cell*s* [30]. As the cells are labeled and allowed to grow in cell growth medium for next 8 days, they divide after labeling and the specific activity (activity per cell) is reduced to the predicted values over a period of 8-day incubation. These observations corroborate data reported by other investigators [25, 30]. Our results also suggest that the cell growth medium containing fetal bovine serum could reduce the labeling efficiency, maybe due to the interference of serum proteins and lipids that could bind the ^89^Zr-oxine complex via transient noncovalent bonding, such as hydrogen bonds, π effect, and hydrophobic bonds. Once labeled, ^89^Zr stably remained associated with live cells.

We optimized our labeling conditions such that CAR-T cells derived from Jurkat cell line and from human peripheral blood derived lymphocytes, which maintained their crucial and functional biological attributes. These attributes were assessed by a matrix of cell-surface and functional biological assays and included CAR-T cell viability, T cell subtype, cell proliferation, retention of radionuclide, chemotaxis, granzyme B, T cell exhaustion, intracellular IFN-gamma expression and specific cell killing of IL-13Rα2 positive target tumor cells. Radiolabeling of CAR-T cells with ^89^Zr-oxime did not affect any of these biological attributes. Both radiolabeled CAR-Jurkat and CAR-T cells retained ≥ 0.5 μCi/10^6^ cell for the next eight days. This amount of activity is within detection limits to detect cells by powerful PET cameras after I.V. infusion in mice. Interestingly, ^89^Zr-oxine labeled CAR-T cells with radioactivity burden did not lose their invasive attributes in Boyden chamber chemotaxis assays, providing additional evidence that the radiolabeling of CAR-T cells with ^89^Zr-oxine did not affect biological functions.

Because interference with cellular and biological functions by radiolabeling with a metal like Zr may cause an inconsistency between the localization of radiolabeled cells detected by imaging techniques and real-time trafficking in vivo, we focused on investigating additional key biological attributes of radiolabeled CAR-T cells. Our in vitro data showed that ^89^Zr-oxine used at the optimized labeling conditions did not induce any increase in apoptotic nuclei, caspase 3/7 and granzyme B activities in post-labeled cells. Similarly, we neither observed any significant change in T cell subtype (CD3 + , CD4 + , CD8 + and scFV-IL-13Rα2 + cell populations), T cell activation phenotype markers expression ( CD28, CD44 and CD69) nor intracellular IFN-γ levels in post-labeled CAR-T cells. Furthermore, the expression of T cell exhaustion markers (PD-1, LAG-3 and TIM3) was unchanged between unlabeled and radiolabeled CAR-T cells. These data indicate that ^89^Zr-oxine radiolabeling has no detrimental effects on cellular functions of CAR-T cells.

Furthermore, ^89^Zr-oxine radiolabeling did not alter the most important feature, potency, of CAR-T cells as assessed by two independent assays, cytotoxicity and IFN-γ release assays. Co-culture of IL-13Rα2 positive and IL-13Rα2 negative glioma cells with varying number of unlabeled and radiolabeled CAR-T cells showed similar cytotoxic activity and released similar amount of IFN-γ. Thus, ^89^Zr-oxine radiolabeling does not affect the potency of CAR-T cells. CAR-T cells, unlike other adoptive T cell products facilitate MHC-independent tumor cell killing by allowing T cells to bind target cell surface antigens, for instance IL-13Rα2 in the present study, through a single-chain variable fragment (scFv) recognition domain. Upon engagement, CAR-T cells form a non-classical immune synapse, required for their effector function [31–33]. These cells then initiate their tumor cell killing effects. Their persistence in the host and functional outputs are tightly dependent on the receptor's individual components-scFv, spacer domain, and costimulatory domains-and how this cellular machinery functions converges to augment CAR T cell performance. In the present study, we also observed that IL-13Rα2 negative glioma cells are not killed by either labeled or unlabeled CAR-T cells.

Our strategy of radiolabeling of CAR-T cells with radio isotope ^89^Zr offer unique advantages to radiolabeled CAR-T cells rapidly and efficiently. Various strategies have been employed in the past to label cells with imaging isotopes for non-invasive in vivo cell tracking for cell-based therapeutic product imaging. More common among these are, ^18^F-FDG (for PET) [34, 35] and ^111^In-oxine (for SPECT) [36]-based imaging analyses. Though ^18^F-FDG is useful for evaluating the delivery of cells and early fate of cells (approximately first few hours), it is not appropriate for in vivo cell tracking after 24 h post-infusion because of its short half-life and poor retention in the cells. Inability of ^18^F-FDG to allow cell tracking beyond 24 h restricts its usefulness in cell-based therapy. For cell therapy based translational studies including adoptive T cell therapy, early engraftment period of first couple of weeks post- cell therapy product infusion is the most critical time period for their biodistribution and trafficking in vivo [37, 38]. Therefore, bio-imaging technologies should be robust over this time frame to allow evaluation of various interventions assessing adverse events and other toxicities. The capability to monitor genetically engineered or modified cells in vivo beyond 24 h is also of high importance for evaluation of biodistribution and trafficking the cellular products using radiolabeled leukocytes. Zr-based compounds or nanoparticles are reported to be safe for human cells and cultured embryonic kidney cells and induce no genotoxicity [39, 40]. ^89^Zr-based radio tracers were shown to be safe when used in a number of pre-clinical studies [41]. Our results are in agreement with other published data, which reported that ^89^Zr-oxine synthesized with different technologies to radiolabel either CTL, NK, DC, bone marrow or stem cells have no interfering effects on cell viability, cytotoxic and functional activity [25, 42–46], which may help in designing more robust and effective biodistribution and cell trafficking studies using PET/CT or PET/MRI technology.

## Conclusions

In conclusion, the ^89^Zr-oxine labeling technique of CAR-T cells we developed is simple, robust and stable allowing CAR-T cells to maintain their critical biological attributes and functions. Collectively, our in vitro data demonstrate that radiolabeled CAR-T cells exhibit similar proliferation, migratory capacity, activation status, potency and specificity to IL-13Rα2 as that of non-labeled cells.

We believe this success will allow us to better use PET-based non-invasive imaging of cell trafficking and biodistribution of CAR-T cells in vivo. To better realize the full potential of our technique, additional studies are planned to assess the trafficking and biodistribution of human CAR-T cells in vivo in animal models of human cancers that are relevant to cell trafficking and biodistribution.

### Supplementary Information

**Additional file 1: Figure S1.** Schematic diagram of scFv-IL-13Rα2 antibody fragment.

**Additional file 2: Table S1.** Comparative Assessment of FACS and IFA Techniques for Phenotype Expression in CAR-T cells.

**Additional file 3: Table S2.** Characterization of subtype of scFv-IL-13Rα2-CAR-T cells after Radiolabeling with ^89^ Zr-oxine on day 7.

## Data Availability

The data analyzed are available from the corresponding author on reasonable request.

## References

[1] Brown CE, Adusumilli PS (2016). Next frontiers in CAR T-cell therapy. Mol Ther Oncolytics.

[2] Brown CE, Alizadeh D, Starr R, Weng L, Wagner JR, Naranjo A, Ostberg JR, Blanchard MS, Kilpatrick J, Simpson J (2016). Regression of glioblastoma after chimeric antigen receptor T-cell therapy. N Engl J Med.

[3] Giavridis T, van der Stegen SJC, Eyquem J, Hamieh M, Piersigilli A, Sadelain M (2018). CAR T cell-induced cytokine release syndrome is mediated by macrophages and abated by IL-1 blockade. Nat Med.

[4] Guedan S, Ruella M, June CH (2019). Emerging cellular therapies for cancer. Annu Rev Immunol.

[5] June CH, O'Connor RS, Kawalekar OU, Ghassemi S, Milone MC (2018). CAR T cell immunotherapy for human cancer. Science.

[6] Kalos M, Levine BL, Porter DL, Katz S, Grupp SA, Bagg A, June CH (2011). T cells with chimeric antigen receptors have potent antitumor effects and can establish memory in patients with advanced leukemia. Sci Transl Med.

[7] Maus MV, June CH (2014). CARTs on the road for myeloma. Clin Cancer Res.

[8] Morgan RA, Dudley ME, Wunderlich JR, Hughes MS, Yang JC, Sherry RM, Royal RE, Topalian SL, Kammula US, Restifo NP (2006). Cancer regression in patients after transfer of genetically engineered lymphocytes. Science.

[9] Ramos CA, Heslop HE, Brenner MK (2016). CAR-T cell therapy for lymphoma. Annu Rev Med.

[10] Sadelain M (2017). CD19 CAR T cells. Cell.

[11] Thomas C, Tampe R (2017). Structure of the TAPBPR-MHC I complex defines the mechanism of peptide loading and editing. Science.

[12] Thomas C, Tampe R (2017). Proofreading of peptide-MHC complexes through dynamic multivalent interactions. Front Immunol.

[13] Batlevi CL, Matsuki E, Brentjens RJ, Younes A (2016). Novel immunotherapies in lymphoid malignancies. Nat Rev Clin Oncol.

[14] Gross G, Waks T, Eshhar Z (1989). Expression of immunoglobulin-T-cell receptor chimeric molecules as functional receptors with antibody-type specificity. Proc Natl Acad Sci U S A.

[15] Ramos CA, Savoldo B, Dotti G (2014). CD19-CAR trials. Cancer J.

[16] Lee DW, Gardner R, Porter DL, Louis CU, Ahmed N, Jensen M, Grupp SA, Mackall CL (2014). Current concepts in the diagnosis and management of cytokine release syndrome. Blood.

[17] Curran KJ, Margossian SP, Kernan NA, Silverman LB, Williams DA, Shukla N, Kobos R, Forlenza CJ, Steinherz P, Prockop S (2019). Toxicity and response after CD19-specific CAR T-cell therapy in pediatric/young adult relapsed/refractory B-ALL. Blood.

[18] Davila ML, Sadelain M (2016). Biology and clinical application of CAR T cells for B cell malignancies. Int J Hematol.

[19] Gill SI, Frey NV, Hexner E, Metzger S, O'Brien M, Hwang WT, Brogdon JL, Davis MM, Fraietta JA, Gaymon A (2022). Anti-CD19 CAR T cells in combination with Ibrutinib for the treatment of chronic lymphocytic leukemia. Blood Adv.

[20] Brooks S, Frey N, Porter D, June C, Lacey S, Bagg A (2016). The cytological features of CAR(T) cells. Br J Haematol.

[21] Frey NV, Porter DL (2016). CAR T-cells merge into the fast lane of cancer care. Am J Hematol.

[22] Kansagra AJ, Frey NV, Bar M, Laetsch TW, Carpenter PA, Savani BN, Heslop HE, Bollard CM, Komanduri KV, Gastineau DA (2019). Clinical utilization of chimeric antigen receptor T-cells (CAR-T) in B-cell acute lymphoblastic leukemia (ALL)-an expert opinion from the European Society for Blood and Marrow Transplantation (EBMT) and the American Society for Blood and Marrow Transplantation (ASBMT). Bone Marrow Transplant.

[23] Melenhorst JJ, Chen GM, Wang M, Porter DL, Chen C, Collins MA, Gao P, Bandyopadhyay S, Sun H, Zhao Z (2022). Decade-long leukaemia remissions with persistence of CD4(+) CAR T cells. Nature.

[24] Singh N, Frey NV, Engels B, Barrett DM, Shestova O, Ravikumar P, Cummins KD, Lee YG, Pajarillo R, Chun I (2021). Antigen-independent activation enhances the efficacy of 4–1BB-costimulated CD22 CAR T cells. Nat Med.

[25] Bansal A, Pandey MK, Demirhan YE, Nesbitt JJ, Crespo-Diaz RJ, Terzic A, Behfar A, DeGrado TR (2015). Novel (89)Zr cell labeling approach for PET-based cell trafficking studies. EJNMMI Res.

[26] Kathirgamanathan P, Surendrakumar S, Antipan-Lara J, Ravichanndaran S, Reddy VR, Ganeshamurugan S, Kumaraveri M, Arkley V, Blake AJ, Bailey D (2011). Discovery of two new phases of zirconium tetrakis(8-hydroxyquinolinolate): synthesis, crystal structure and their electron transporting characteristics in organic light emitting diodes (OLEDs). J Mater Chem.

[27] Joshi BH, Plautz GE, Puri RK (2000). Interleukin-13 receptor alpha chain: a novel tumor-associated transmembrane protein in primary explants of human malignant gliomas. Cancer Res.

[28] Lichtenfels R, Biddison WE, Schulz H, Vogt AB, Martin R (1994). CARE-LASS (calcein-release-assay), an improved fluorescence-based test system to measure cytotoxic T lymphocyte activity. J Immunol Methods.

[29] Neri S, Mariani E, Meneghetti A, Cattini L, Facchini A (2001). Calcein-acetyoxymethyl cytotoxicity assay: standardization of a method allowing additional analyses on recovered effector cells and supernatants. Clin Diagn Lab Immunol.

[30] Sato N, Wu H, Asiedu KO, Szajek LP, Griffiths GL, Choyke PL (2015). (89)Zr-Oxine complex PET cell imaging in monitoring cell-based therapies. Radiology.

[31] Benmebarek MR, Karches CH, Cadilha BL, Lesch S, Endres S, Kobold S (2019). Killing mechanisms of chimeric antigen receptor (CAR) T cells. Int J Mol Sci.

[32] Csaplar M, Szollosi J, Gottschalk S, Vereb G, Szoor A (2021). Cytolytic activity of CAR T cells and maintenance of their CD4+ subset is critical for optimal antitumor activity in preclinical solid tumor models. Cancers (Basel).

[33] Kiesgen S, Messinger JC, Chintala NK, Tano Z, Adusumilli PS (2021). Comparative analysis of assays to measure CAR T-cell-mediated cytotoxicity. Nat Protoc.

[34] Stojanov K, de Vries EF, Hoekstra D, van Waarde A, Dierckx RA, Zuhorn IS (2012). [18F]FDG labeling of neural stem cells for in vivo cell tracking with positron emission tomography: inhibition of tracer release by phloretin. Mol Imaging.

[35] Zhang Y, Dasilva JN, Hadizad T, Thorn S, Kuraitis D, Renaud JM, Ahmadi A, Kordos M, Dekemp RA, Beanlands RS (2012). (18)F-FDG cell labeling may underestimate transplanted cell homing: more accurate, efficient, and stable cell labeling with hexadecyl-4-[(18)F]fluorobenzoate for in vivo tracking of transplanted human progenitor cells by positron emission tomography. Cell Transplant.

[36] Roca M, de Vries EF, Jamar F, Israel O, Signore A (2010). Guidelines for the labelling of leucocytes with (111)In-oxine. Inflammation/infection taskgroup of the European Association of Nuclear Medicine. Eur J Nucl Med Mol Imaging..

[37] Naumova AV, Balu N, Yarnykh VL, Reinecke H, Murry CE, Yuan C (2014). Magnetic resonance imaging tracking of graft survival in the infarcted heart: iron oxide particles versus ferritin overexpression approach. J Cardiovasc Pharmacol Ther.

[38] Naumova AV, Modo M, Moore A, Murry CE, Frank JA (2014). Clinical imaging in regenerative medicine. Nat Biotechnol.

[39] Demir E, Burgucu D, Turna F, Aksakal S, Kaya B (2013). Determination of TiO2, ZrO2, and Al2O3 nanoparticles on genotoxic responses in human peripheral blood lymphocytes and cultured embyronic kidney cells. J Toxicol Environ Health A.

[40] Demir E, Turna F, Vales G, Kaya B, Creus A, Marcos R (2013). In vivo genotoxicity assessment of titanium, zirconium and aluminium nanoparticles, and their microparticulated forms, in Drosophila. Chemosphere.

[41] Kurebayashi Y, Choyke PL, Sato N (2021). Imaging of cell-based therapy using (89)Zr-oxine ex vivo cell labeling for positron emission tomography. Nanotheranostics.

[42] Asiedu KO, Ferdousi M, Ton PT, Adler SS, Choyke PL, Sato N (2018). Bone marrow cell homing to sites of acute tibial fracture: (89)Zr-oxine cell labeling with positron emission tomographic imaging in a mouse model. EJNMMI Res.

[43] Beckford Vera DR, Smith CC, Bixby LM, Glatt DM, Dunn SS, Saito R, Kim WY, Serody JS, Vincent BG, Parrott MC (2018). Immuno-PET imaging of tumor-infiltrating lymphocytes using zirconium-89 radiolabeled anti-CD3 antibody in immune-competent mice bearing syngeneic tumors. PLoS ONE.

[44] Mali SS, Shim CS, Kim H, Patil PS, Hong CK (2016). In situ processed gold nanoparticle-embedded TiO2 nanofibers enabling plasmonic perovskite solar cells to exceed 14% conversion efficiency. Nanoscale.

[45] Tavare R, Escuin-Ordinas H, Mok S, McCracken MN, Zettlitz KA, Salazar FB, Witte ON, Ribas A, Wu AM (2016). An effective immuno-PET imaging method to monitor CD8-dependent responses to immunotherapy. Cancer Res.

[46] Weist MR, Starr R, Aguilar B, Chea J, Miles JK, Poku E, Gerdts E, Yang X, Priceman SJ, Forman SJ (2018). PET of adoptively transferred chimeric antigen receptor T cells with (89)Zr-oxine. J Nucl Med.

