# Correction: Characterization of chimeric antigen receptor modified T cells expressing scFv-IL-13Rα2 after radiolabeling with ^89^Zirconium oxine for PET imaging

**DOI:** 10.1186/s12967-023-04496-7

**Published:** 2023-09-19

**Authors:** Pamela Leland, Dhiraj Kumar, Sridhar Nimmagadda, Steven R. Bauer, Raj K. Puri, Bharat H. Joshi

**Affiliations:** 1 Tumor Vaccines and Biotechnology Branch, Division of Cellular and Gene Therapies, Office of Tissues and Advance Therapies, Center for Biologics Evaluation and Research, Food and Drug Administration, 10903 New Hampshire Avenue, Silver Spring, MD 20993 USA; 2 Department of Radiology and Radiological Science, Johns Hopkins University School of Medicine, Baltimore, MD USA; 3 Present Address: Wake Forest Institute of Regenerative Medicine, Winston Salem, NC USA; 4 Present Address: Iovance Biotherapeutics, San Carlos, CA USA

**Correction: Journal of Translational Medicine (2023) 21:367** 10.1186/s12967-023-04142-2

Following publication of the original article [1], we have been notified that the legend order of X axis in Fig. 7 was incorrect. The corrected x-axis legend Fig. 7 is given below:

**Fig. 7:**
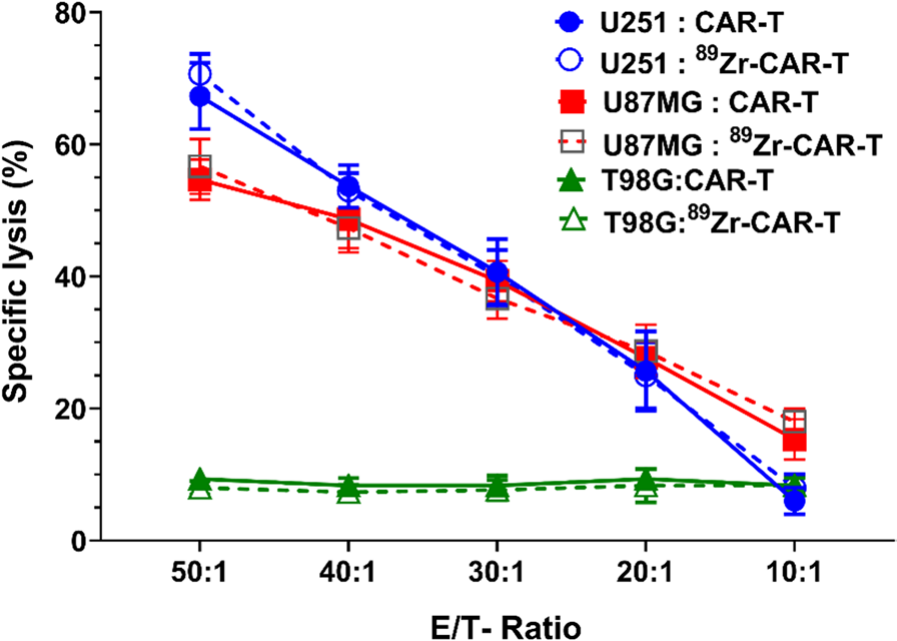
^89^Zr-oxine radiolabeling did not interfere with the biological functional potency of CAR-T cells. **A** In vitro potency assay of radiolabeled and unlabeled CAR-T cells showed no significant difference in their IL-13Rα2 positive target tumor cell killing abilities in a co-culture assay as described in Materials and Methods. **B** Co-culture assay of labeled and unlabeled CAR-T effector cells with IL-13Rα2 positive U251, A172 and U87MG target cells showed similar values of IFN-γ release in 20-h culture. IL-13Rα2 negative T98G glioma cells co-cultured with labeled and unlabeled CAR-T cells secreted basal amounts of IFN-γ. A representative data of three independent experiments performed in quadruplicate is shown
