# Supplementary figures and images for: Characterization of chimeric antigen receptor modified T cells expressing scFv-IL-13Rα2 after radiolabeling with 89Zirconium oxine for PET imaging
Source: J Transl Med. 2023 Jun 7;21:367. doi: 10.1186/s12967-023-04142-2 (PMC10246418; doi:10.1186/s12967-023-04142-2)

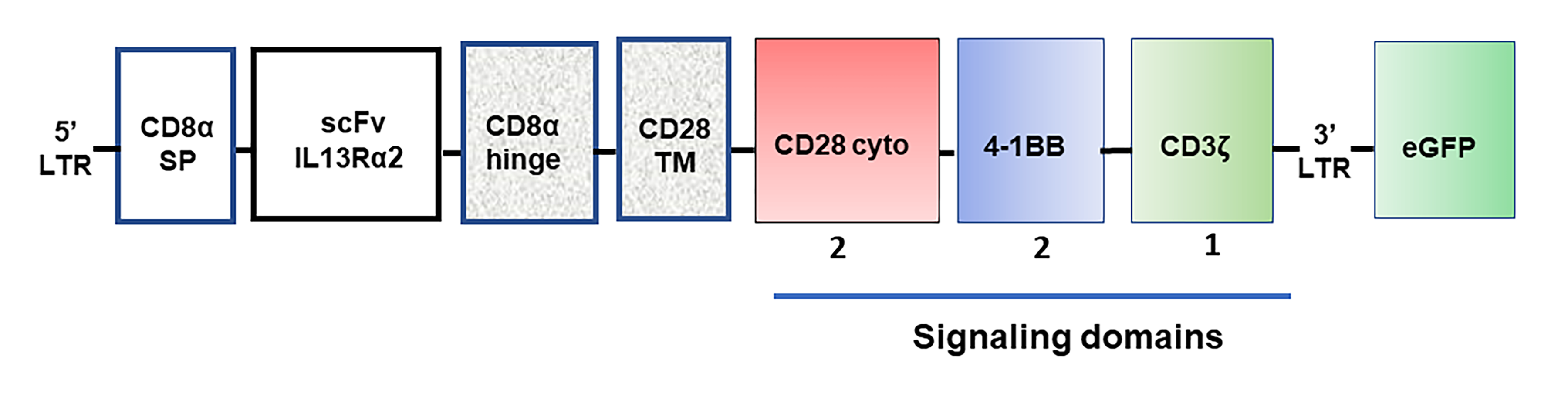

Supplement: Supplementary file 1 — Additional file 1: Figure S1. Schematic diagram of scFv-IL-13Rα2 antibody fragment. [file 12967_2023_4142_MOESM1_ESM.tif]
