# Supplementary material for: Characterization of chimeric antigen receptor modified T cells expressing scFv-IL-13Rα2 after radiolabeling with 89Zirconium oxine for PET imaging
Source: J Transl Med. 2023 Jun 7;21:367. doi: 10.1186/s12967-023-04142-2 (PMC10246418; doi:10.1186/s12967-023-04142-2)
Supplement: Supplementary file 2 — Additional file 2: Table S1. Comparative Assessment of FACS and IFA Techniques for Phenotype Expression in CAR-T cells. [file 12967_2023_4142_MOESM2_ESM.docx]

Supplementary Table 1:

Comparative Assessment of FACS and IFA Techniques for Phenotype Expression in CAR-T cells

_____________________________________________________________________________________

Phenotype % Positive Cells*

------------------------------------------------------------------------------------------

FACS IFA

------------------------------------- ------------------------------------

CD3+ 94.5 ± 4.5 93.2 ± 5.7

CD4+ 60.7 ± 5.5 62.5 ± 6.2

CD8+ 26.4 ± 1.8 28.7 ± 2.6

______________________________________________________________________* = Each value is a mean ± SD of four independent experiments performed in quadruplicate.

** = Concordance between two methods = 94.28 ± 4.2%
