# Supplementary material for: Characterization of chimeric antigen receptor modified T cells expressing scFv-IL-13Rα2 after radiolabeling with 89Zirconium oxine for PET imaging
Source: J Transl Med. 2023 Jun 7;21:367. doi: 10.1186/s12967-023-04142-2 (PMC10246418; doi:10.1186/s12967-023-04142-2)
Supplement: Supplementary file 3 — Additional file 3: Table S2. Characterization of subtype of scFv-IL-13Rα2-CAR-T cells after Radiolabeling with 89 Zr-oxine on day 7. [file 12967_2023_4142_MOESM3_ESM.docx]

Supplementary Table 2:

Characterization of subtype of scFv-IL-13Rα2-CAR-T cells after Radiolabeling with ^89^ Zr-oxine on day 7

____________________________________________________________________________________

Phenotype % Positive Cells*

----------------------------------------------------------------------------------------

Unlabeled CAR-T cells Radiolabeled CAR-T cells

------------------------------- ---------------------------------------

CD4+ 56.7 ± 4.5 54.5 ± 5.2

CD8+ 35.4 ± 2.1 38.7 ± 3.1

CD3+ 93.4± 4.2 94.0 ± 5.6

scFv-IL-13Rα2+ 48.2 ± 3.2 49.6 ± 5.3

____________________________________________________________________________* = Each value is a mean ± SD of four independent experiments performed in quadruplicate using specified antibody
